# Possible adaptation measures for climate change in preventing heatstroke among older adults in Japan

**DOI:** 10.3389/fpubh.2023.1184963

**Published:** 2023-09-22

**Authors:** Marie Fujimoto, Katsuma Hayashi, Hiroshi Nishiura

**Affiliations:** Kyoto University School of Public Health, Kyoto, Japan

**Keywords:** emergency transportation, heatstroke, risk reduction, climate change, statistical model

## Abstract

**Introduction:**

Heatstroke mortality is highest among older adults aged 65 years and older, and the risk is even doubled among those aged 75 years and older. The incidence of heatstroke is expected to increase in the future with elevated temperatures owing to climate change. In the context of a super-aged society, we examined possible adaptation measures in Japan that could prevent heatstroke among older people using an epidemiological survey combined with mathematical modeling.

**Methods:**

To identify possible interventions, we conducted a cross-sectional survey, collecting information on heatstroke episodes from 2018 to 2019 among people aged 75 years and older. Responses were analyzed from 576 participants, and propensity score matching was used to adjust for measurable confounders and used to estimate the effect sizes associated with variables that constitute possible interventions. Subsequently, a weather-driven statistical model was used to predict heatstroke-related ambulance transports. We projected the incidence of heatstroke-related transports until the year 2100, with and without adaptation measures.

**Results:**

The risk factor with the greatest odds ratio (OR) of heatstroke among older adults was living alone (OR 2.5, 95% confidence interval: 1.2–5.4). Other possible risk factors included an inability to drink water independently and the absence of air conditioning. Using three climate change scenarios, a more than 30% increase in the incidence of heatstroke-related ambulance transports was anticipated for representative concentration pathways (RCP) 4.5 and 8.5, as compared with a carbon-neutral scenario. Given 30% reduction in single living, a 15% reduction in the incidence of heatstroke is expected. Given 70% improvement in all three risk factors, a 40% reduction in the incidence can be expected.

**Conclusion:**

Possible adaptation measures include providing support for older adults living alone, for those who have an inability to drink water and for those without air conditioning. To be comparable to carbon neutrality, future climate change under RCP 2.6 requires achieving a 30% relative reduction in all three identified risks at least from 2060; under RCP 4.5, a 70% reduction from 2050 at the latest is needed. In the case of RCP 8.5, the goal of heatstroke-related transports approaching RCP 1.9 cannot be achieved.

## 1. Introduction

Heatstroke is an environmentally induced condition caused by exposure to a very warm environment and an inability to lower elevated body temperature (1). Depending on the mechanism of development, heatstroke is divided into classic heatstroke, caused purely owing to environmental conditions, and exertional heatstroke, induced by physical exercise (2). People who have difficulty adapting to a warm environment, including older adults and those people with chronic illnesses, are more likely to develop heatstroke (3–6). Morbidity of heatstroke is elevated with a transient increase in temperature such as heatwaves (7, 8). To reduce mortality, preventing heatstroke is more effective than treatment, involving simple yet realistic countermeasures to reduce heatstroke incidence (9). Published preventive measures of heatstroke include the installation of air-conditioners (10) and enhancement of public support for older adults (11).

The incidence of heatstroke is expected to increase in the future with rising temperatures owing to climate change. The Intergovernmental Panel on Climate Change has set a goal of limiting the increase in the global average temperature to 1.5°C by the end of the 21st century, as a mitigation measure (12). Among health problems associated with climate change, heatstroke is a disease for which measures to reduce risk are required worldwide (13). Health-related risk assessment of climate change has taken place under various scenarios of temperature increase across the world (13–20), and possible risk reduction via adaptation measures to climate change has been explored in recent years (21, 22). Population aging is also reported to increase the burden of heat-related health risks under climate change (23, 24), and heatstroke mortality is known to be highest among people aged 65 years and older, and the risk is even doubled among those aged 75 years and older.

In Japan, the government Ministry of the Environment (MOE) has taken the initiative to inform the public regarding the risk of heatstroke, using the wet bulb globe temperature (WBGT) as a standard indicator (25). The WBGT is classified into five discrete categories: less than 21°C, 21°C–25°C, 25°C–28°C, 28°C–31°C, and 31°C or higher (26). When the temperature exceeds 28°C, warnings are issued by the government via mass media. Despite various countermeasures, approximately 1000 annual deaths owing to heatstroke have been reported in Japan in recent years, and more than 80% of heatstroke deaths are among people over 65 years of age [(27); Supplementary material 1]. To consider prevention strategies of heatstroke-related deaths in Japan, a super-aged society, studies have been conducted using various statistical models (28, 29). We proposed a forecasting model using the maximum daily WBGT under several climate change scenarios (30). However, intervention studies have been limited to date.

The purpose of the present study was to identify possible adaptation measures among older adults in Japan in the context of a super-aged society and to estimate their effectiveness in preventing heatstroke. Identifying possible adaptation measures can help assist various stakeholders, including local governments, community caregivers and so on to consider future preparedness plans to mitigate the risk of heatstroke even under changing climate. Such contingency plan may decrease the disease burden and mortality of heatstroke. In this study, we first conducted a cross-sectional epidemiological survey to identify possible risk factors via survey and then modeled what is the potential that decrease in these risk factors could have in the future in preventing heatstroke. We also used a climate-driven prediction model to predict heatstroke-related ambulance transports under various climate change scenarios.

## 2. Materials and methods

### 2.1. Identification of risk factors

#### 2.1.1. Cross-sectional survey

We carried out a cross-sectional epidemiological online survey among Japanese residents with family members or other relatives aged 75 years or older. We focused on this group, because the risk of heatstroke among people aged 75 years or older is known to be twice as high as that among people aged 65 years or older [(27); Supplementary material 1]. Participants were selected non-randomly from a list of registered users of a Japanese internet research company called Mellinks Ltd. Respondents did not receive remunerations, but upon completion of survey, they received local “points” that could be exchanged for valuable goods via the company. The internet-based survey was carried out from September 14 to 24, 2021, by navigating respondents to visit the website with questionnaire. The questionnaire was designed based on published studies (3–6), and we focused on heatstroke episodes from 2018 to 2019. Heatstroke episode was defined in our survey based on criteria adapted from the ‘Heatstroke Treatment Guidelines 2015’ (31) and a reference (1) which are known to have been comprehensive even among non-medical experts. A more detailed description is provided in Supplementary material 2. We specifically surveyed 2018–2019, because of retrospective nature of our study, and also to avoid the potential impact of the coronavirus disease 2019 (COVID-19) pandemic on the results of the questionnaire. Moreover, socioeconomic level and comorbidities were also surveyed in indirect manners. Not only exploring the presence of air conditioner, the survey questions included gender and the number of household occupants that are known to influence socioeconomic levels of life among older people (32). As for comorbidities, we investigated whether there were any pre-existing comorbidities that are associated with the risk of heatstroke, including depression, heart failure, hypertension, kidney diseases, and Parkinson’s disease. A version of our questionnaire translated into English is available in Supplementary material 2.

#### 2.1.2 Statistical analyses

The dichotomous (2-category) outcome was an episode of heatstroke from 2018 to 2019, and we investigated univariate and multivariate associations of explanatory variables with the occurrence of heatstroke episodes. First, we investigated the univariate statistical association between heatstroke and explanatory variables, estimating the odds ratio (OR) as the effect size measure. For the calculation of OR, we used a univariate logistic regression. Subsequently, among variables that were significantly associated with heatstroke in the univariate analysis, we selected variables into which we can intervene. To adjust for potential confounders among measured variables, one-to-one propensity score matching was carried out for each factor into which we expected to intervene (33). A logistic regression model was used to estimate propensity scores, involving four measured variables (i.e., age, sex, underlying comorbidities, and inability to move to a cooler place during hot weather). Using a caliper width with a propensity score standard deviation of 0.2, matching was performed using nearest-neighbor matching and non-replacement methods. The balance of baseline variables between the two propensity-matched groups was examined using standardized differences, and more than 10% was considered unbalanced, following the convention of matching procedure (34). Using the same propensity score, we conducted a sensitivity analysis with the inverse probability of treatment weighting (IPTW) method.

### 2.2. Future prediction scenario of heatstroke

#### 2.2.1. Data source for prediction model

Three pieces of data in Tokyo were used: (i) the number of heatstroke patients transported by ambulance (35), (ii) daily maximum WBGT (25), and (iii) weather data from observatories (36). Data on the number of daily transported patients aged 65 years and older are routinely collected by the Fire and Disaster Management Agency (FDMA) from May to September each year. The FDMA data only shows a dichotomous age group indicating whether heatstroke patient is 65 years and older, not in the form of individual age of heatstroke patient. Two other datasets were obtained using publicly available data from the referenced source and collected during the FDMA collection period. To calibrate our model, all these datasets were prepared for the period of 5 years from 2015 to 2019. Climatological variables including WBGT from weather station data were used to predict the number of heatstroke-related ambulance transports. WBGT values were dealt with in the same manner as the unit of temperature, i.e., °C. WBGT values during the abovementioned 5 years represent direct measurements in Tokyo.

In this study, future climatological variables were obtained from climate change scenarios based on the Coupled Model Intercomparison Project Phase 6 published by the National Institute for Environmental Studies (NIES) (37). Three scenarios were extracted from NIES: (i) Model for Interdisciplinary Research on Climate version 6 (MIROC6), (ii) Meteorological Research Institute Earth System Model version 2.0 (MRI-ESM-2.0), (iii) the Institute Pierre-Simon Laplace climate mode (IPSL-CM6A-LR). We specifically examined these three scenarios because the carbon-neutral scenario is available as part of the representative concentration pathways (RCP). Future meteorological data were collected at RCP 1.9, 2.6, 4.5, and 8.5 by specifying the latitude and longitude of the weather stations in Tokyo, among which RCP 1.9 corresponds to a carbon-neutral scenario. Future WBGT by the year 2100 was calculated using meteorological data with an estimator developed by Ono et al. (38). To calculate the risk at population level, past demographic data were obtained from the 2015 census (39), and data in the future were extracted from the Climate Change Adaptation Information Platform (40).

#### 2.2.2. Projection model

In our previous study (30), projections were made using a forecasting model that uses daily maximum WBGT. Letting *T_d_* be the daily maximum WBGT on day *d*, the expected number of heatstroke-related transports was modeled as:


(1) $$E\left(n\left(T_{d}\right)\right)=\{\begin{array}{c} \beta \;\mathrm{for}\;T_{d}<T_{w}\\ \beta exp\left(r\;\left(T_{d}-T_{w}\right)\right)\;\mathrm{for}\;T_{w}\geq T_{d} \end{array}$$

where *Tw* is the WBGT threshold (e.g., 28°C) above which the dose–response increase in heatstroke is seen, *β* is the constant risk at WBGT below *Tw*, and r is the rate of risk increase as a function of WBGT. We demonstrated the usefulness of WBGT in projection, but projection using a simplistic model was unable to capture observed heatstroke counts when the temperature was greatly elevated for several consecutive days.

We thus attempted to improve the equation in the present study, additionally accounting for weather data related to heat (i.e., global solar radiation and a sequence of hot days) in the prediction. Because our earlier model was unable to capture the heatstroke count during heatwaves that continued for several days, heat acclimation was also considered (i.e., before and after natural adaptation was taken into account), which is in line with a published study (41). Dealing with global solar radiation (*s_d_*) (kW/m^2^), and similarly dealing with WBGT on *d* (*u_d_*) as dichotomous (whether the daily maximum WBGT exceeded 31°C for 2 or 3 consecutive days), we modeled the daily number of heatstroke-related ambulance transports as


(2) $$E\left(n\left(T_{d}\right)\right)=\{\begin{array}{c} \beta_{0}\;\mathrm{for}\;T_{d}<T_{w,0},\mathrm{before adaptation}\\ \beta_{1}\;\mathrm{for}\;T_{d}<T_{w,1},\mathrm{after adaptation}\;\\ \begin{array}{l} \beta_{0}\exp\left(r_{0}\;\left(T_{d}-T_{w,0}\right)+\gamma_{1,0}s_{d}+\gamma_{2,0}u_{d}\right)\\ \;\mathrm{for}\;T_{w,0}\geq T_{d},\mathrm{before adaptation} \end{array}\\ \begin{array}{l} \beta_{1}\exp\left(r_{1}\;\left(T_{d}-T_{w,1}\right)+\gamma_{1,1}s_{d}+\gamma_{2,1}u_{d}\right)\\ \;\mathrm{for}\;T_{w,1}\geq T_{d},\mathrm{after adaptation}\; \end{array} \end{array}$$

where parameters *β* and *r*, as well as the threshold value of WBGT *T*_*w*,_ were assumed to be varying via heat acclimation (subscript 0 denotes before adaptation and 1 denotes after adaptation) and *γ_1,i_* and γ_2,i_ are coefficients for *s_d_* and *u_d_*, respectively, before (*i* = 0) and after (*i* = 1) natural adaptation representing the average daily temperature (°C) reached the highest value of the season. This particular model was identified as most reasonably capturing observed heatstroke-related ambulance transports (Fujimoto et al., under review). Assuming that the number of ambulance transports owing to heatstroke follows a Poisson distribution, maximum likelihood estimation was performed to obtain optimal parameter values. The Akaike information criterion (AIC) was computed and the model with the best fit was selected.

The best fit model was used for projection using weather data of RCP 1.9, 2.6, 4.5, and 8.5 in three climate change scenarios to yield the predicted number of heatstroke-related transports from 2020 to 2100. The number of heatstroke cases was calculated per 100,000 people.

Because the heatstroke incidence is greatly affected by temperature variations in each year, a 5-year arithmetic average was taken for each 5-year period. Relative risk per year was computed, comparing projections against the empirically observed 5-year median from 2015 to 2019 and the carbon-neutral scenario in the same year (RCP 1.9).

### 2.3. Intervention effectiveness

The *per capita* probability of heatstroke from May to September in Tokyo was empirically estimated as ranging from 0.12 to 0.08%; thus, we judged the incidence of heat stroke to be rare, and we adopted odds ratios as an approximation of risk ratio. To calculate the effect of intervention measures in reducing the incidence of heatstroke, we used the adjusted OR of factor *v*, *q_v_* and the proportion of older adults having the risk factor *v* in year *t*, *p_v,t_*. Among the population at risk with factor *v*, we observed *q_v_p_v,t_* as the risk of heatstroke; among the remainder without factor *v*, the population at risk is $1-p_{v,t}$ . Normalizing these, the fraction of heatstroke that occurs among people with factor *v* would be $q_{v}p_{v,t}/\left(q_{v}p_{v,t}+\left(1-p_{v,t}\right)\right)$ . Similarly, the fraction of heatstroke among people without risk factor *v* would be $\left(1-p_{v,t}\right)/\left(q_{v}p_{v,t}+\left(1-p_{v,t}\right)\right)$ . Of these, in the presence of interventions, only the $q_{v}p_{v,t}$ part of the numerator would be reduced by intervening the risk factor *v* for a fraction *i_v,t_* in year *t*. That is, at the population level, the relative risk reduction by intervening factor *v* by *i_v,t_* is:


(3) $$\;k_{v}=\frac{q_{v}p_{v,t}\left(1-i_{v,t}\right)+\left(1-p_{v,t}\right)}{q_{v}p_{v,t}+\left(1-p_{v,t}\right)}\;$$

where *k_v_* is the relative decrease in the number of heatstroke patients attained by intervention into risk factor *v.* Because we handled multiple risk factors, we calculated the projected number of heatstroke-related ambulance transports under interventions, $n^{\prime }\left(T_{d}\right)$ as


(4) $$E\left(\underset{d}{\sum }n^{\prime }\left(T_{d}\right)\right)=\underset{v}{\prod }k_{v}\times \underset{d}{\sum }E\left(n\left(T_{d}\right)\right)\;$$

That is, the expected number of heatstroke-related transports per 100,000 population estimated for the period from May to September was obtained by multiplying the obtained preventive effect *k*_v_ for all examined risk factors. Computation was carried out, assuming that adaptation measures are implemented from 2030 and that it would take 5 years from 2030 to reach the plateaued level of intervention.

To calculate the future proportion of people with pre-determined risk factors among people aged 65 years and older (i.e., to calculate $p_{v,t}$ ), the following analyses were conducted. Due to data limitation of the FMDA’s heatstroke transport data, which only specifies whether patients were 65 years and older, we calculated age-specific risk based on this age grouping. First, the projected rate of older adults living alone by 2040 was retrieved from the National Institute of Population and Social Security Research in Tokyo (42), and the estimate was used as empirical data for additional future projections. Because the size of the entire population of Japan will decrease (with deaths of the baby boomer generation), with a substantial decrease in the demand for older adult care, a quadratic equation was fitted to capture the forthcoming decline in the proportion of older people living alone and was fitted to the abovementioned data to 2040. Alternatively, in the case of a scenario in which the proportion of the older population living alone remains constant, a cubic exponential formula was used. As for the proportion of people who are unable to drink water independently, the proxy value was the percentage of those certified as having care need level 3 or more (i.e., a condition that requires total assistance in the activities of daily living) (43), retrieved from the Tokyo Metropolitan Government (44). Information on certification rates by sex and age group for care need levels 3, 4, and 5 for the years 2015–2020 were used; it was assumed that the care need level was determined by age and will not change after 2020. The age-dependent proportion of older adults with care levels 3–5 in 2020 was used to project the proportion of people who are unable to drink water independently through 2100 (40). Lastly, the percentage of households without air-conditioning was estimated using the observed percentage from 2011 to 2022 from the National Survey of Living Conditions (45) in Japan conducted by the Ministry of Health, Labour, and Welfare.

All calculations were performed using JMP statistical software, version 16.0 (SAS Institute Inc., Cary, NC, United States) and R software version 4.2.0 (The R Project for Statistical Computing, Vienna, Austria).

## 3. Results

### 3.1. Explanatory variables of heatstroke risk

The cross-sectional survey involved 576 participants, including 166 older adults with a history of heatstroke and 410 without a heatstroke history. Participants’ characteristics and the results of univariate analysis are summarized in Table 1. Among explanatory variables of heatstroke episodes, (i) male sex, (ii) having an underlying medical condition, and (iii) living alone were significant. The OR and 95% confidence interval (CI) of these variables was 1.7 (95% CI: 1.2, 2.4), 2.5 (95% CI: 1.6, 3.8), and 2.1 (95% CI: 1.2, 3.5), respectively. Although not significant, the ORs of inability to drink water independently and absence of air-conditioning were 1.5 (95% CI: 1.0, 2.3) and 1.6 (95% CI: 0.9, 2.6), respectively.

**Table 1:** Characteristics of participants with crude odds ratio, confidence intervals, and *p*-values for heatstroke.

Characteristic	Participants with heatstroke episode(s), *N* = 166^1^	Non-heatstroke participants, *N* = 410^1^	Odds ratio (95% confidence interval)	*p*-value
Age (years)	84.4 (7.8)	86.7 (6.9)	0.95 (0.9, 1.0)	<0.01
Gender (Male)	92/166 (55%)	174/410 (42%)	1.7 (1.2, 2.4)	<0.01
Underlying medical condition	53/166 (32%)	65/410 (16%)	2.5 (1.6, 3.8)	<0.01
Require nursing care	134/166 (81%)	301/410 (73%)	1.5 (1.0, 2.4)	0.08
Inability to move	55/166 (33%)	151/410 (37%)	0.9 (0.6, 1.2)	0.46
Living alone	28/166 (17%)	37/410 (9.0%)	2.1 (1.2, 3.5)	0.01
Inability to drink water	38/166 (23%)	68/410 (17%)	1.5 (1.0, 2.3)	0.10
Absence of air-conditioner	27/166 (16%)	45/410 (11%)	1.6 (0.9, 2.6)	0.11

1 Mean (standard deviation); n/N (%).

Underlying medical conditions included four diseases (depression, heart failure, kidney disease, and Parkinson’s disease) as these potentially lead to the inability to move if exacerbated. Requiring nursing care refers to the need for help in activities of daily living (e.g., eating and dressing). The inability to move describes whether an older adult is able to move to a cooler place in elevated temperatures. Inability to drink water indicates that the person is unable to drink water independently.

Then, factors into which interventions could be made were further examined. Based on the results from univariate analysis, three intervention-related factors were (i) people living alone, (ii) being unable to drink water independently, and (iii) not having an air-conditioner. After propensity score matching and IPTW calculations, Table 2 shows the adjusted ORs for these factors: 2.5 (95% CI: 1.2, 5.4), 1.2 (95% CI: 0.7, 2.1), and 1.6 (95% CI: 0.8, 3.2), respectively. Although the 95% CIs from propensity score matching were widened compared with the results univariate analysis, IPTW analysis yielded significant results for all three variables (Table 2). Accordingly, we examined the effects of intervention for all three factors in a subsequent analysis. Supplementary Tables S1–S3 show the results of propensity score matching.

**Table 2:** Odds ratio of developing heatstroke.

Risk factors	Crude OR^1^) (95% CI^2^)	Adjusted OR^1^ (95% CI)	
		PS^3^-matched	IPTW^4^
Living alone	2.1 (1.2, 3.5)	2.5 (1.2, 5.4)	2.1 (1.6, 2.7)
Inability to drink water	1.5 (1.0, 2.3)	1.2 (0.7, 2.1)	2.0 (1.5, 2.7)
Absence of air-conditioner	1.6 (0.9, 2.6)	1.6 (0.8, 3.2)	1.6 (1.2, 2.0)

OR^1^, odds ratio; CI^2^, confidence interval; PS^3^, propensity score; IPTW^4^, inverse probability of treatment weighting.

Directed acyclic graphs and confirmed variables are presented in Supplementary Figure S1. A logistic regression model with six baseline independent variables (age, sex, underlying disease, inability to move, and two other factors) was used to estimate the propensity score.

### 3.2. Future prediction scenario of heatstroke

In analyzing multiple models describing heatstroke-related ambulance transports from 2015 to 2019, Supplementary Table S4 shows the summary of model comparisons (including AIC values and mean squared error). The best fit model was identified as:


(5) $$E\left(n\left(T_{d}\right)\right)=\{\begin{array}{c} \beta_{0}\;for\;T_{d}<T_{w,0},beforeadaptation\\ \beta_{1}\;for\;T_{d}<T_{w,1},afteradaptation\;\\ \begin{array}{l} \beta_{0}\exp\left(r_{0}\;\left(T_{d}-T_{w,0}\right)+\gamma_{1}s_{d}+\gamma_{2}u_{d}\right)\;\\ for\;T_{w,0}\geq T_{d},beforeadaptation \end{array}\\ \begin{array}{l} \beta_{1}\exp\left(r_{1}\;\left(T_{d}-T_{w,1}\right)+\gamma_{1}s_{d}+\gamma_{2}u_{d}\right)\;\\ for\;T_{w,1}\geq T_{d},afteradaptation. \end{array} \end{array}$$

**Figure 1:**
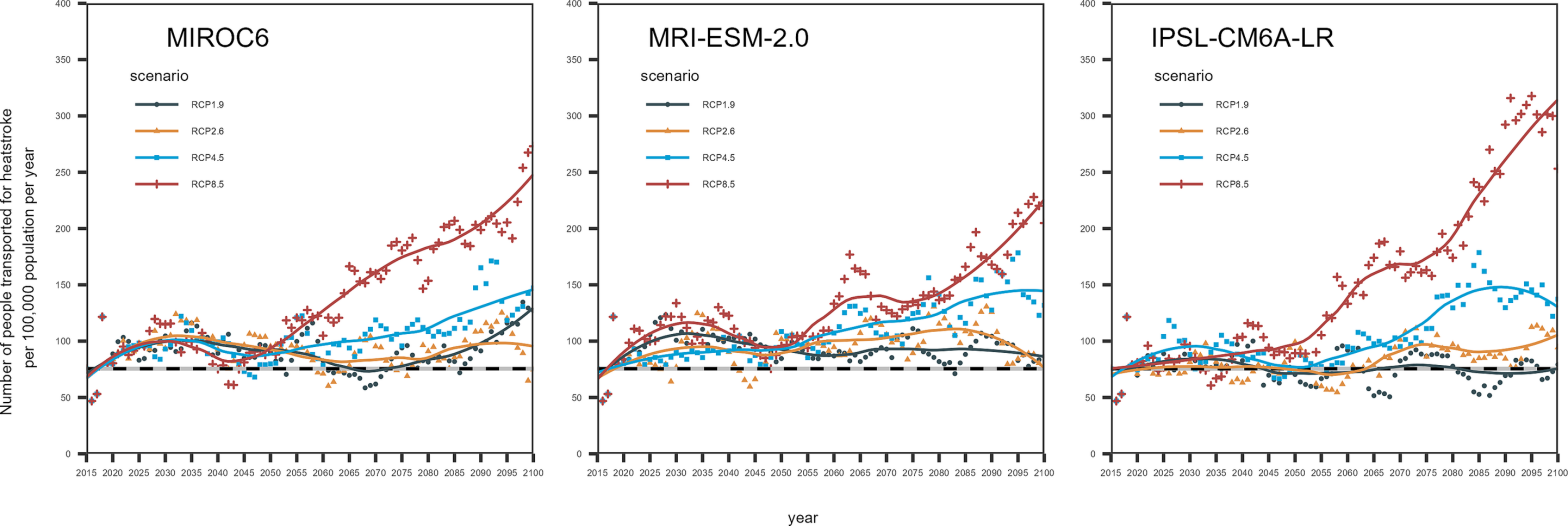
Projected number of heatstroke-related ambulance transports among older adults in Tokyo from 2015 to 2100 using three climate change scenarios (MIROC6, MRI-ESM-2.0, and IPSL-CM6A-LR). The vertical axis is the number of heatstroke-related ambulance transports per 100,000 population, and the horizontal axis represents the year. The dots are the 5-year average number of heatstroke-related transports per 100,000 population per year; the line represents the smoothed line. Smoothing was done using the LOESS method, with a span of 0.5. The colors of the dots and lines are the same for each RCP. The black dotted line at the bottom of the image shows the 5-year median since 2015 for the number of people transported by ambulance owing to heatstroke.

Compared with equation (2), it should be noted that γ_1_ and γ_2_ in equation (5) do not change before and after natural adaptation owing to the average daily temperature reaching the highest value of the season. The variable *u_d_* indicates whether there were 3 consecutive days with the daily maximum WBGT exceeding 31°C. Maximum likelihood estimates of the parameter were estimated at *T_w,0_* = 22.1, *T_w,1_* = 19.3, *r_0_* = 0.31, *r_1_* = 0.34, *β_0_* = 0.87, *β_1_* = 0.22, *γ_1_* = 0.04, and *γ_2_* = 0.58, respectively. Using these parameters, projection scenarios of heatstroke were produced by the year 2100 for each RCP using three climate change scenarios, MIROC6, MRI-ESM-2.0, and IPSL-CM6A-LR. The predicted results are shown in Figure 1. Compared with the 5-year median number of heatstroke-related ambulance transports from 2015, even RCP 1.9 (i.e., carbon-neutral scenario) was projected to involve increased heatstroke-related transports under MIROC6 and MRI-ESM-2.0. Specifically, the MIROC6 model projected a maximum increase of 40%, while the MRI-ESM-2.0 model projected a maximum increase of 30% in heatstroke-related transports. Although there were differences depending on climate change scenarios, RCP 4.5 and RCP 8.5 showed increments in the number of heatstroke-related ambulance transports among people aged over 65 years compared with projections from RCP 1.9.

The results of relative risk calculations are shown in Table 3. Taking the baseline as the 5-year median from 2015 for the period from May to September, the number of heatstroke-related ambulance transports were increased for RCP 2.6, 4.5, and 8.5. Depending on the year, the number of heatstroke-related transports will increase by approximately 20% with RCP 2.6, 30% with RCP 4.5, and 50% with RCP 8.5. In particular, we found that after 2060, the relative increase compared with the baseline period will continue to exceed 50% with RCP 8.5. Using RCP 1.9 as a baseline, RCP 2.6 yielded an approximate 5–20% increase after 2060. In MIROC6, compared with the RCP 1.9 scenario, RCP4.5 climate scenario was expected to increase the number of heatstroke-related ambulance transport by more than 30% compared to RCP1.9, and RCP8.5 scenario more than 50%, in the second half of the 21st century.

**Table 3:** Relative risk of the increase in heatstroke-related ambulance transports relative to 5-year median from 2015 to 2019 and carbon-neutral scenario.

Relative risk of increase in heatstroke-related ambulance transports										
Scenario	Baseline		2030s	2040s	2050s	2060s	2070s	2080s	2090s	2100
MIROC6	2015–19	RCP2.6	31.7 (19.4, 39.1)	20.1 (0, 29.1)	19.6 (0, 22.0)	1.8 (0, 27.6)	6 (0, 21.7)	14.8 (0.4, 30.3)	31.9 (0, 39.7)	0		RCP4.5	25.3 (15.0, 38.2)	5.3 (0, 26.7)	24.6 (5.4, 38.4)	20.8 (4, 31.7)	32.5 (21.2, 36.5)	31.3 (27.6, 48.8)	45.6 (36.3, 55.9)	48.7		RCP8.5	24.3 (5.0, 34.7)	5.4 (0, 19.2)	36.3 (14.3, 40.8)	48.6 (28, 54.6)	57.2 (48.5, 60.6)	60.9 (50.8, 63.5)	63.3 (60.5, 71.8)	72.4	RCP1.9	RCP2.6	6.2 (0, 26.8)	5.0 (0, 19.8)	2.8 (0, 22.5)	6.9 (0, 41.9)	3.5 (0, 35.1)	10.3 (0, 20.5)	3.7 (0, 16)	0		RCP4.5	1.4 (0, 23.7)	0 (0, 10.3)	6.8 (0, 31.5)	26.1 (0, 45.2)	25.1 (1.9, 47.9)	25.7 (9.6, 39.7)	15.6 (0, 44.7)	13.7		RCP8.5	0 (0, 18.3)	0 (0, 5.5)	14.0 (3.4, 40.5)	53.0 (21.3, 62.4)	54.2 (43.6, 61.3)	54.2 (47, 63.8)	48.6 (39.5, 54.1)	53.5
MRI-ESM-2.0	2015–19	RCP2.6	29.2 (1.8, 39.5)	4.1 (0, 29.5)	24.3 (17.4, 28.7)	26.3 (13.6, 37.1)	23.7 (9.8, 42.2)	34.1 (23.1, 42.2)	14.2 (5.1, 40.0)	2.1		RCP4.5	16.2 (10.0, 27.0)	15.5 (3.2, 27.7)	22.9 (11.4, 30.5)	36.6 (27.3, 42.4)	38.2 (22.6, 51.7)	41.8 (28.1, 51.3)	48.5 (38.6, 57.7)	42.7		RCP8.5	35.7 (22.7, 43.5)	21.6 (0, 38.4)	27.3 (19.6, 31.0)	48.7 (36.5, 57.3)	42.7 (38.1, 47.6)	53.1 (44.7, 61.6)	63.1 (52.7, 66.9)	63.1	RCP1.9	RCP2.6	6.8 (0, 15.3)	0 (0, 23.8)	7.7 (3.7, 17.6)	14.1 (0, 27.7)	4.4 (0, 34.5)	19.5 (11.1, 33.7)	0.9 (0, 17.0)	0		RCP4.5	0 (0, 3.8)	0 (0, 13.4)	7.9 (0, 22.1)	25.4 (9.2, 37.0)	13.3 (0, 43.3)	31.5 (23.8, 42.2)	38.5 (18.1, 53.6)	34.3		RCP8.5	11.6 (0, 24.6)	0 (0, 16.1)	11.9 (3.6, 19.6)	40.0 (22.7, 53.4)	24.1 (16.7, 40.6)	44.6 (38.9, 54)	54.9 (37.5, 65)	57.7
IPSL-CM6-LR	2015–19	RCP2.6	7.4 (0, 22.9)	0 (0, 19.7)	0 (0, 23.9)	3.3 (0, 20.0)	22.2 (12.4, 32.2)	14.3 (8.3, 22.2)	24.0 (1.9, 33.6)	20.1		RCP4.5	15.5 (0, 28.2)	5.8 (0, 15.6)	4.7 (0, 13.9)	23.6 (19.4, 30)	29.4 (20.7, 46.3)	49.6 (41.7, 57.7)	46.0 (38.1, 49.9)	45		RCP8.5	0 (0, 26.6)	23.4 (10.9, 34.9)	22.3 (0.1, 51.9)	54.7 (43.3, 59.9)	54.2 (51.7, 61.3)	67.2 (56.6, 72)	74.9 (73.6, 76.2)	70.2	RCP1.9	RCP2.6	3.9 (0, 21.1)	8.0 (0, 20)	2.1 (0, 31.9)	23.6 (0, 40.3)	9.0 (0, 22)	29.0 (19.3, 41.5)	22.3 (0, 40.7)	16.1		RCP4.5	8.3 (0, 28.6)	4.1 (0, 31.9)	11.6 (0, 27.3)	38.0 (2.2, 47.2)	18.8 (7.9, 38.2)	57.5 (40, 70.5)	44.8 (40.2, 56)	42.3		RCP8.5	0 (0, 8.4)	24.1 (9.3, 41.1)	35.0 (20.8, 44.3)	61.8 (31.0, 71.7)	46.4 (44.2, 55.5)	73.8 (55.3, 80.8)	75.0 (71.9, 77.9)	68.7

RCP, representative concentration pathways.

Numbers in the table are 10-year median and range (minimum to maximum). There is no range for 2100 (there is a predicted number only for this particular year). RCP 1.9 is the climate scenario in which carbon neutrality is achieved; higher values after RCP are considered to result in greater temperature increases. The 5-year average of the number of heatstroke transports per 100,000 population for each year with RCP 2.6, RCP 4.5, and RCP 8.5 was calculated. The difference compared with the baseline for each year was then calculated, and the ratio of each year in the number of changes was calculated. As practiced in excess risk evaluation, the difference was taken over the course of the year for the data point at which the predicted value exceeded the baseline value. The units are percentages.

### 3.3. Future prediction of intervention effectiveness

**Figure 2:**
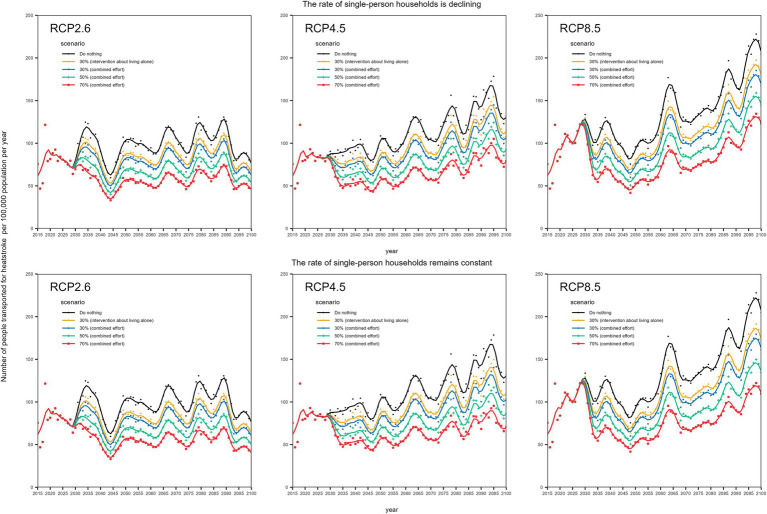
Projected effectiveness of interventions against heatstroke in Tokyo this figure shows the predicted results for heatstroke-related ambulance transports without and with intervention (adaptive policy) for each RCP, using MRI-ESM-2.0 in Tokyo. The dots represent the 5-year average number of heatstroke-related transports among older adults transported in each year, and the lines represent smoothing lines. LOESS was used as the smoothing method, with a span of 0.5. We assumed that the target will be achieved over a 5-year period starting in 2030. The top three panels show the effects of adaptation measures per RCP for a scenario in which the future proportion of older adults living alone declines along with the population from 2040. The bottom three panels show the effects of adaptation measures per RCP for a scenario in which the future proportion of older adults living alone is maintained constant. Combined effort means all three risk factors were assumed to be intervened into (i.e., living alone, inability to drink water independently, and absence of air conditioning).

For the intervention scenarios, we calculated heatstroke-related ambulance transports, assuming a relative decrease in risk groups of 30% (i.e., 30% relative decrease in the number of older adults living alone) and similarly, relative decreases of 30, 50, 70, 90, and 100% for all three risk factors. Figure 2 shows the expected baseline number of heatstroke-related ambulance transports with two different future outcomes for the proportion of older people living alone (i.e., declining or remaining constant), along with results of the abovementioned interventions (i.e., adaptation policies). Figure 2 shows the results using MRI-ESM-2.0; the results with the two other climate change scenarios are shown in Supplementary Figures S2, S3 (Supplementary material 3).

Under a scenario in which the proportion of older people living alone declines over time in the future, a 30% relative reduction in the number of older adults living alone would result in an up to 15% decrease in the number of heatstroke-related transports. Similarly, concerted interventions (i.e., averting all three risks) would result in a 20% decrease in heatstroke by decreasing the proportions with risk factors at 30%. These findings were similar in another scenario where the future proportion of older adults living alone was maintained constant (Figure 2). In RCP 8.5, even with 100% relative reduction in all identified risks, the frequency of heatstroke could still not be lowered in comparison with the carbon-neutral scenario in some years.

## 4. Discussion

Using daily maximum WBGT values, additional meteorological information, and accounting for probable heat acclimatization during consecutive hot days in the summer season, we estimated the number of heatstroke-related ambulance transports in the future under various climate change scenarios. The proposed model can provide better fit to observed data than our earlier model (30), yielding a long-term prediction in Tokyo until the year 2100. We showed that to reduce the future burden of heatstroke below historical levels, heatstroke adaptation measures are vital, even with a carbon-neutral scenario. To be comparable to carbon neutrality, with future climate change under RCP 2.6, a 30% relative reduction in all three identified risks from 2060 is required, and under RCP 4.5, a 70% relative reduction from 2050 is needed. In the case of RCP 8.5, even a 100% reduction is not comparable to RCP 1.9 in some years, calling for serious mitigation measures.

To the best of our knowledge, our study is the first to combine an epidemiological survey and future projection of heatstroke in the context of adaptation measures. Although a few excellent machine learning-based predictions of heatstroke-related ambulance transports in Japan have been conducted (28, 29) and epidemiological studies of admitted patients with heatstroke have been reported (44, 45), no studies have examined risk factors of the onset of heatstroke, aiming to reduce this risk. Although our survey was cross-sectional, the snapshot survey of heatstroke history among older adults enabled us to cover the risk of broad-spectrum heatstroke (including mild cases), allowing for the calculation of ORs. Intervenable factors of heatstroke were found to be (i) living alone, (ii) inability to drink water independently, and (iii) the absence of air-conditioning. The adjusted OR allowed us to examine possible future scenarios under which the above risk factors were partially improved via social support (as part of a future adaptation policy). With an elevated risk of heatstroke in the future, intervenable factors (i)–(iii) above could alleviate the heatstroke risk in the future such that the number of cases can be maintained to a number comparable to a carbon-neutral scenario.

For the calculation of future interventions, obtaining adjusted ORs is key. Although the present study was cross-sectional, propensity score matching allowed us to adjust for observed measurable confounders. Among examined variables that can be intervened into, living alone yielded the highest OR value. Among all examined variables, having an underlying medical condition yielded the highest risk estimate (followed by living alone), but having a medical condition is not intervenable. Thus, not merely adjusting for confounders but also using the matching method was useful to adjust for known strong predictors. Considering that older adults tend to have difficulty in recognizing and objectively judging heat levels (5, 6), having peer or professional support, especially for people living alone, is deemed a reasonable option.

Classically, potential interventions among older adults have been restricted to the use of air-conditioning and frequent drinking of water to prevent dehydration (1, 3, 48), which is important, as dehydration can frequently develop into heatstroke. However, the effect sizes of lack of air-conditioning and an inability to drink water were smaller than that of living alone. The greater importance of living alone poses a challenge for adaptation measures because living with others cannot be achieved via peer support only and calls for concerted action by local governments. Japanese older adults generally have lower incomes than working-age adults, with the main income from pensions, and single-person households are expected to have lower incomes than multiple-person households (32). These difficulties often lead to older adults having multiple risk factors, including a lack of air-conditioning, especially older adults who live alone.

There are four limitations of the present study. First, we cannot exclude the possibility of unadjusted confounders during propensity score matching and IPTW. We systematically searched for published studies and drew directed acyclic graphs, but using the selected survey and modeling method, we cannot exclude the presence of unmeasured confounders. Second, the sample size might have been small to sufficiently identify risk factors via propensity score matching. To ensure the representativeness, we checked the correlation of (i) the proportion of older adults aged 75 years and older and (ii) the participants per population size across prefectures, and the resulting R^2^ being 0.94 reflects the fact that older people were geographically balanced in their sampling frequency. A larger sample size with additional variables is needed in future studies. Third, estimated ORs were retrieved from the survey of people aged 75 years and older; the actual population-based estimate for people aged 65 years and older may be smaller than our calculation. Thus, discussions over more precise policy-related goals require similar surveys addressing this point. Fourth, some parameters were retrieved for all of Japan whereas the proposed model was restricted to Tokyo. This model specifically captures the situation in Tokyo, which, despite having one of the lowest proportions of older adult people in Japan, still records one of the highest numbers of heatstroke incidents per year. Japan, with its elongated geography from north to south, has diverse summer temperature environments across its regions. However, it’s well known that a dose–response relationship exists between the daily maximum WBGT and the number of heatstroke cases in Japan all prefectures (49). Given the availability of similar data like this study, it could be possible to apply this model to other regions of Japan. Nevertheless, differences in regional factors such as the urban heat island effect and population characteristics demand caution when generalizing these findings. Further studies are needed to identify risk factors for all of Japan and to develop a representative prediction model using different geographic and temporal settings.

Despite these limitations, we successfully estimated the future number of heatstroke-related ambulance transports using climate change scenarios in Japan. We found that even with a 70% relative reduction in all identified risk factors under RCP 2.6, 4.5, and 8.5, the resulting relative decrease in heatstroke would be approximately 40%. Even if carbon neutrality were achieved, we estimated that the number of ambulance transports owing to heatstroke would exceed the 5-year median in 2015. Aiming to achieve carbon neutrality as the temporary goal, it is advisable to implement adaptation measures to reduce the risk of heatstroke among older adults.

## 5. Conclusion

The number of heatstroke-related ambulance transports among people aged 65 years and older in Tokyo was projected through 2100 under various climate change scenarios. In a cross-sectional survey, intervenable factors for heatstroke were shown to be (i) living alone, (ii) inability to drink water independently, and (iii) absence of air-conditioning, and we estimated their effect sizes. To reduce the future burden of heatstroke below historical levels, heatstroke adaptation measures are vital, even in a carbon-neutral scenario. To be comparable to carbon neutrality, future climate change under RCP 2.6 would require a 30% relative reduction in all three identified risks from 2060, and RCP 4.5 would require a relative reduction of 70% or more from 2050. In the case of RCP 8.5, even a 100% reduction would not be comparable to RCP 1.9, calling for serious mitigation measures. If aiming to achieve carbon neutrality as the temporary goal, it is advisable to implement adaptation measures to reduce the risk of heatstroke among older adults. Based on our findings, a variety of stakeholders can smoothly consider future preparedness plans. For instance, local government could help establish a system that identifies a household at high risk of heatstroke in older people, prioritizing tailor-made interventions for those at risk as part of mitigation strategy (50). Furthermore, these insights could also assist community caregivers and senior citizens themselves to properly understand the forthcoming risk and potentially mitigate future heatstroke risks.

## Data availability statement

Publicly available datasets were analyzed in this study. This data can be found here: original data, including modeled climatological data for the period 2015–2019 and empirical data on heat-related ambulance transports, are openly shared as online supporting material (32–34, 36, 37).

## Ethics statement

Informed consent was obtained via internet from all participants in the cross-sectional survey. After the completion of the survey, Mellinks Ltd. collected and anonymized data in such a way that it could not be traced back to any personally identifiable information. The cross-sectional survey was approved by the Ethics Committee, Kyoto University Graduate School and Faculty of Medicine (no. R3120). The study complied with the Declaration of Helsinki, 2013.

## Author contributions

MF: data curation, formal analysis, investigation, methodology, visualization, writing – original draft, and writing – review and editing. KH: methodology, supervision, and writing – review and editing. HN: conceptualization, methodology, formal analysis, supervision, writing – review and editing, and project administration. All authors contributed to the article and approved the submitted version.

## Funding

This study was supported by the Environment Research and Technology Development Fund (JPMEERF20S11804) of the Environmental Restoration and Conservation Agency of Japan. MF received funding from Kyoto University Medical Student and Researcher Support-Fund, the JSPS KAKENHI (23KJ1228). KH received funding from the JPPS KAKENHI (23K09712). HN received funding from Health and Labor Sciences Research Grants (20CA2024, 20HA2007, 21HB1002, 21HA2016, and 22HA1005), the Japan Agency for Medical Research and Development (JP20fk0108140, JP20fk0108535, JP21fk0108612, and JP23fk0108685), the JSPS KAKENHI (21H03198 and 22K19670), the Japan Science and Technology Agency SICORP program (JPMJSC20U3 and JPMJSC2105), and the RISTEX program for Science of Science, Technology and Innovation Policy (JPMJRS22B4). The funders had no role in the study design, data collection and analysis, decision to publish, or preparation of the manuscript.

## Conflict of interest

The authors declare that the research was conducted in the absence of any commercial or financial relationships that could be construed as a potential conflict of interest.

## Publisher’s note

All claims expressed in this article are solely those of the authors and do not necessarily represent those of their affiliated organizations, or those of the publisher, the editors and the reviewers. Any product that may be evaluated in this article, or claim that may be made by its manufacturer, is not guaranteed or endorsed by the publisher.
