# Supplementary material for: Possible adaptation measures for climate change in preventing heatstroke among older adults in Japan
Source: Front Public Health. 2023 Sep 22;11:1184963. doi: 10.3389/fpubh.2023.1184963 (PMC10556232; doi:10.3389/fpubh.2023.1184963)
Supplement: Supplementary file 2 [file Data_Sheet_2.DOCX]

Supplementary Material 2

Scenario analysis of potential adaptation measures for climate change in preventing heatstroke among older adults in Japan

**Marie Fujimoto, Katsuma Hayashi and Hiroshi Nishiura***

***** Corresponding author: nishiura.hiroshi.5r@kyoto-u.ac.jp

**Questionnaire on heatstroke for older people in an epidemiological study**

This survey is part of a study to collect information on the background of heatstroke in older people. The study is a means of measuring heatstroke in Japan.

The purpose of this study is to identify factors that predispose people aged 75 years or more to heatstroke.

All responses will be handled in a privacy-protected manner. The results of this survey may be reused for research purposes, while ensuring anonymity.

This survey is open to anyone with a family member or relative who is 75 years of age or older as of 2018–2019 and who usually needs assistance in daily life, regardless of whether you live with or apart from the family member or relative. “Assistance in daily life” refers to any or all of the following: dressing, eating, toileting, and bathing, and includes simple assistance levels.

Definitions

A: Heatstroke

Both of the following two conditions should be met.

1. Owing to a hot environment, which leads to a high body temperature, the individual finds it hard to move, has convulsions, or loses consciousness.

2. The individual does not recover easily from the symptoms described above and medical services are required or a physician diagnoses heatstroke. Medical services means providing an IV drip, cooling the body, and so on.

B: Relatives

The survey target's spouse or a relative within the fourth degree of kinship (cousin or closer)."

**Questionnaire on heatstroke in people aged more than 75 years**

Q1. Select one family member or relative over age 75 years. How old is that person?

Q2. What is the sex of the person?

- Male
- Female

Q3. Did the person experience heatstroke during 2018–2019?

- Yes
- No

If Yes: go to Question 4

If No: go to Question 6

Q4. Please select all months in which the person experienced heatstroke during 2018–2019? Multiple responses are possible.

- Jan
- Feb
- Mar
- Apr
- May
- Jun
- Jul
- Aug
- Sept
- Oct
- Nov
- Dec

Q5. Please select all medical care the person received when they were diagnosed with heatstroke. If they had multiple occurrences of heatstroke during 2018–2019, please select the most serious event.

- Received a home visit
- Visited a hospital
- Hospitalized for more than 1 day

Q6. Did the person you selected in Question 1 live with you or your family during 2018–2019?

- Yes
- No

If Yes: go to Question 9

If No: go to Question 7

Q7. What kind of dwelling did the person you selected live in?

- House or apartment
- Nursing home with no nurse in the facility
- Nursing home or hospital with nurses on site during the day

If you chose House or apartment: go to Question 8

For all other responses: go to Question 9

Q8. Did the person you selected in Question 1 live with someone during 2018–2019?

- Yes
- No

Q9. Did the person you selected need someone’s support for dressing, eating, toileting, and bathing?

- Yes
- No

Q10. Did the person you selected have long-term care certification during 2018–2019?

- Support level 1
- Support level 2
- Care level 1
- Care level 2
- Care level 3
- Care level 4
- Care level 5
- Not certified

Q11. Did the person you selected in Question 1 have any other diseases? Multiple responses are possible.

- Depression
- Heart failure
- Hypertension
- Kidney disease
- Parkinson’s disease
- None of the above

Q12. Was the person able to drink water independently?

- Yes
- No

Q13. Was the person able to move to a cooler place by themselves?

- Yes
- No

Q14 Did the person have air-conditioning?

- Yes
- No
- If Yes: go to Question 15
- If No: go to Question 16

Q15. Was the person able to turn on the air-conditioner themselves?

- Yes
- No

Q16. How often did the person’s family check on their condition?

- Once every few hours
- Once every half day
- Once a day
- At intervals of more than once a day

Q17. How often did medical or nursing staff check on the person’s medical condition?

- Once every few hours
- Once every half day
- Once a day
- At intervals of more than once a day
