# Supplementary material for: Possible adaptation measures for climate change in preventing heatstroke among older adults in Japan
Source: Front Public Health. 2023 Sep 22;11:1184963. doi: 10.3389/fpubh.2023.1184963 (PMC10556232; doi:10.3389/fpubh.2023.1184963)
Supplement: Supplementary file 3 [file Data_Sheet_3.DOCX]

Supplementary Material 3

Scenario analysis of potential adaptation measures for climate change in preventing heatstroke among older adults in Japan

**Marie Fujimoto, Katsuma Hayashi and Hiroshi Nishiura***

***** Corresponding author: nishiura.hiroshi.5r@kyoto-u.ac.jp

# Supplementary Figures and Tables

# **Supplementary Figure**s


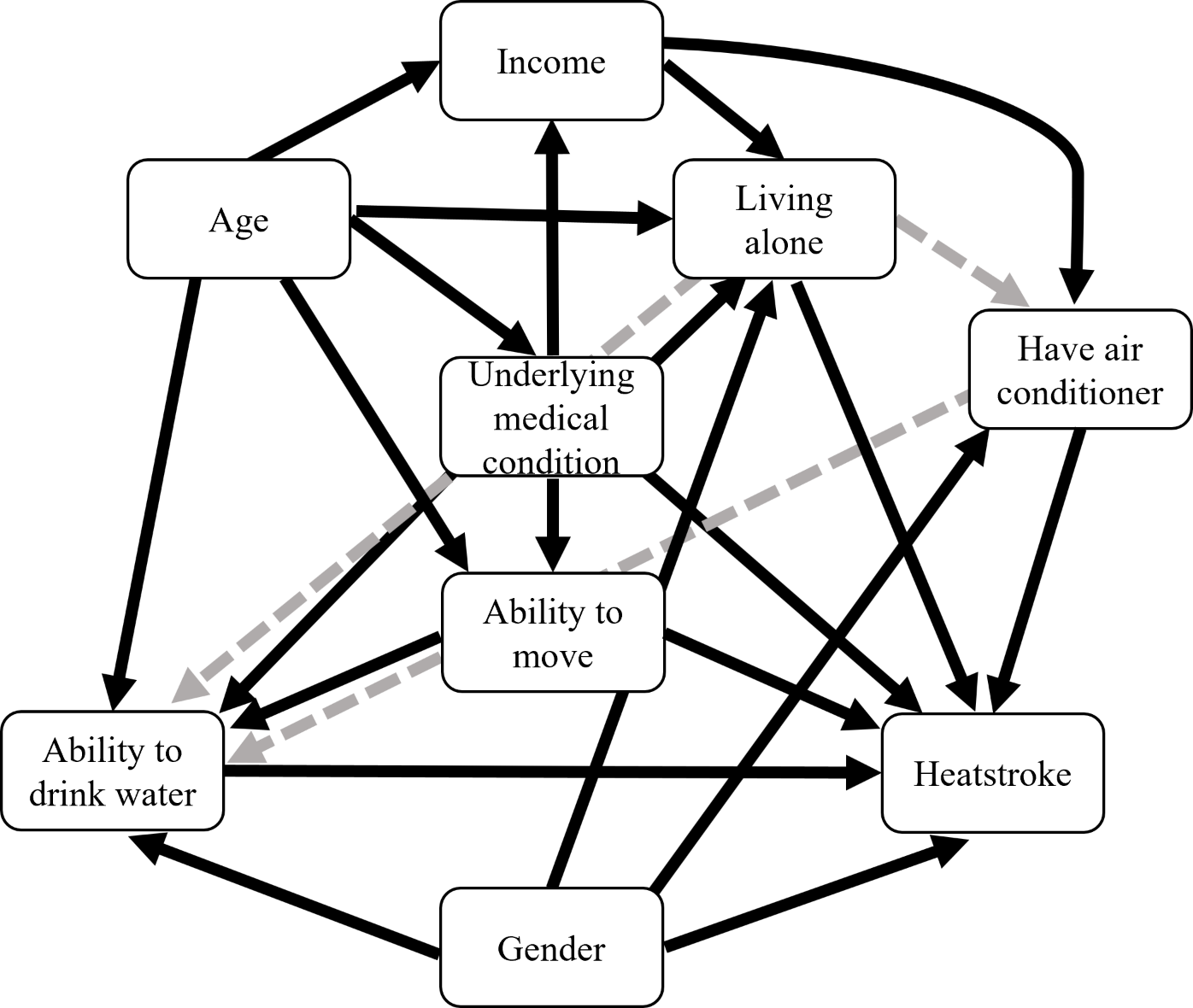


**Supplementary Figure S1.** Causal diagrams for heatstroke in this study

We defined the outcome as the onset of heatstroke, and risk factors were living alone, inability to drink water independently, and not having an air-conditioner. Because intervention of the three risk factors influence each other, we considered that the gray dotted arrows vary according to the intervention factors. Income is an unmeasured confounder in this study. We determined whether to include the other two risk factors as covariates by calculating the mean squared error.


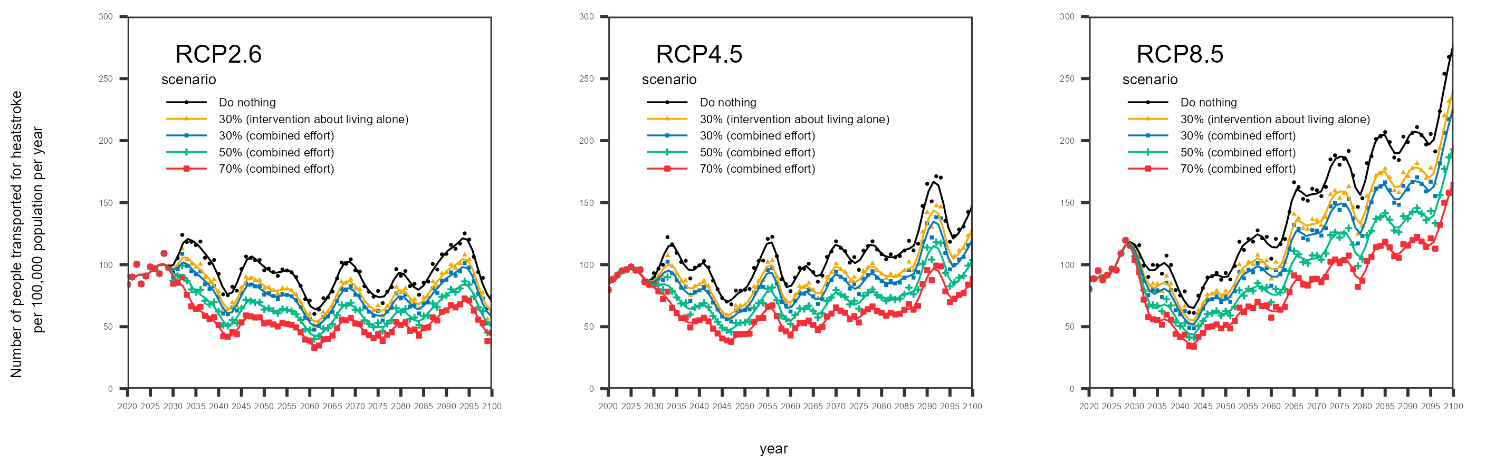


**Supplementary Figure S2. Projected effect of heatstroke adaptation measures using MIROC6.**

Projection of the number of heatstroke-related ambulance transports each year is shown, using data obtained from MIROC6. The dots represent the 5-year average number of heatstroke-related transports among older adults in each year, and the lines represent smoothing lines. LOESS was used as the smoothing method, with a span of 0.5. We assumed that the target will be achieved over a 5-year period starting in 2030. This figure shows the effects of adaptation measures per RCP for a scenario in which the number of older adults living alone declines along with the population after 2040.


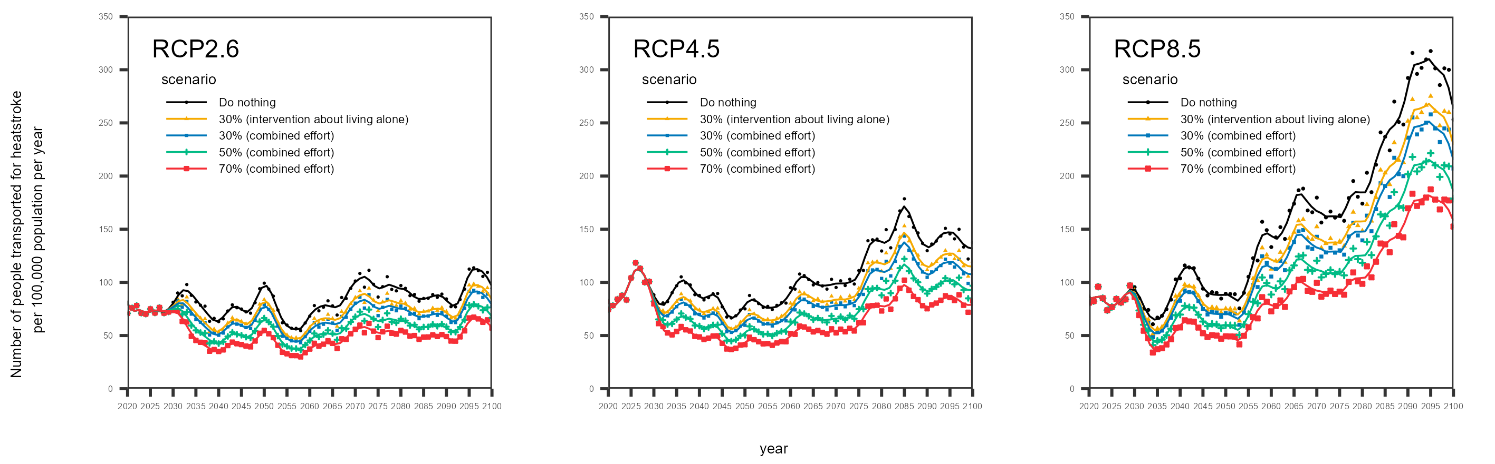


**Supplementary Figure S3. Projected effect of heatstroke adaptation measures using IPSL-CM6-LR**

Projection of the number of heatstroke-related ambulance transports each year is shown, using data obtained from IPSL-CM6-LR. The dots are the 5-year average number of heatstroke-related transports among older adults in each year; the lines are smoothing lines. LOESS was used as the smoothing method, with a span of 0.5. We assumed that the target will be achieved over a 5-year period starting in 2030. This figure shows the effects of adaptation measures per RCP for a scenario in which the number of older adults living alone declines along with the population after 2040.


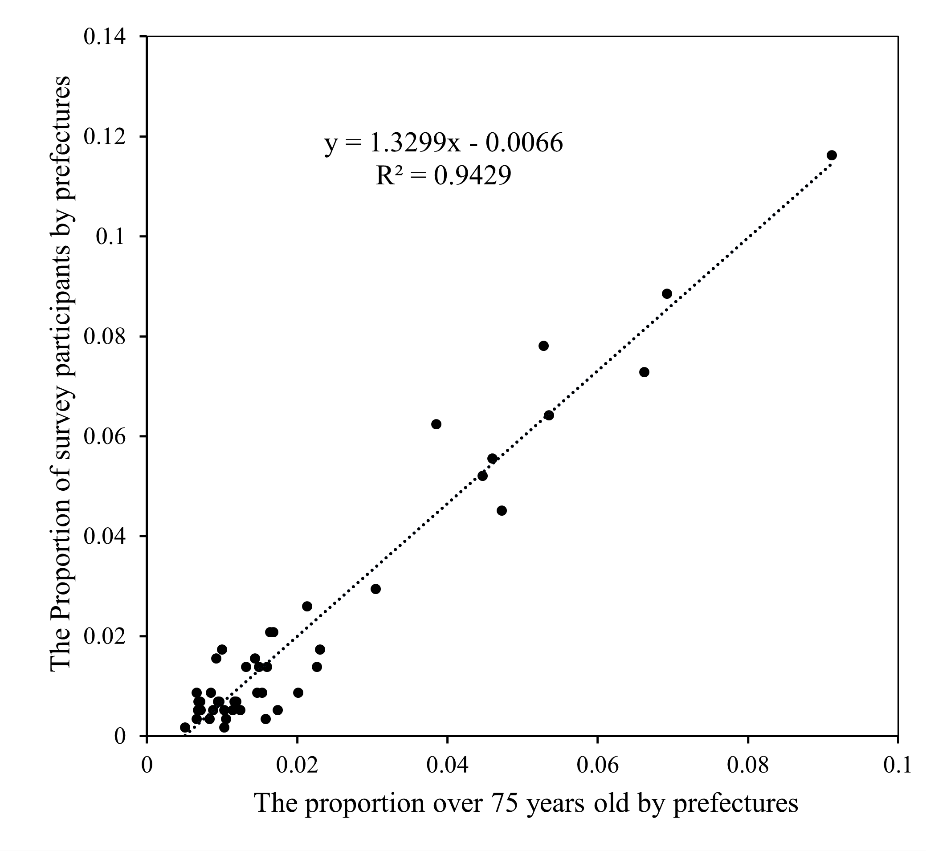


**Supplementary Figure S4. Survey Respondent Proportions and Japanese Population Correlation**

Correlations between the proportion of those surveyed and the proportion of Japan's population aged 75 and older in 46 prefectures. Only Nagano prefecture had no participants. The population was extracted from the 2020 census data.

# Supplementary Tables

**Supplementary Table S1.** **Results from propensity score matching for older adults living alone.**

|  | Unmatched groups | |  | Matched groups | |  |
| --- | --- | --- | --- | --- | --- | --- |
|  | Living together | Living alone |  | Living together | Living alone |  |
|  | n =511 | n = 65 | SD (%) | n = 65 | n =65 | SD (%) |
| Age (years) | 86.1 (7.3) | 85.6 (6.6) | 7.2 | 85.7 (6.9) | 85.6 (6.6) | 1.5 |
| Gender(male) | 240 (47%) | 26 (40%) | 14.1 | 25 (38%) | 26 (40%) | 3.2 |
| Underlying medical condition | 104 (20%) | 14 (22%) | 2.9 | 12 (18%) | 14 (22%) | 7.7 |
| Inability to move | 194 (38%) | 22 (18%) | 8.6 | 10 (15%) | 12 (18%) | 8.2 |
| Inability to drink water | 97 (19%) | 9 (14%) | 13.9 | 8 (12%) | 9 (14%) | 4.6 |
| Absence of air conditioner | 59 (12%) | 13 (20%) | 23.4 | 10 (15%) | 13 (20%) | 12.1 |

We used a logistic regression model with six baseline independent variables (age, sex, underlying disease, inability to move, inability to drink water independently, and absence of air-conditioning) to estimate propensity score. The receiver operating characteristic was 0.65, and the E-value was 2.6. For the calculation of E-values, see (1).

**Supplementary Table S2. Results of propensity score matching for inability to drink water independently.**

|  | Unmatched groups | |  | Matched groups | |  |
| --- | --- | --- | --- | --- | --- | --- |
|  | able to drink water | unable to drink water |  | able to drink water | unable to drink water |  |
|  | n = 410 | n = 106 | SD (%) | n = 102 | n = 102 | SD (%) |
| Age (years) | 86.3 (7.2) | 84.8 (7.2) | 20.8 | 85.3 (7.5) | 85.1 (7.2) | 2.7 |
| Gender(male) | 220 (47%) | 46 (43%) | 20.6 | 50 (49%) | 45 (44%) | 9.8 |
| Underlying medical condition | 95 (20%) | 23 (22%) | 3.5 | 25 (25%) | 21 (21%) | 9.4 |
| Inability to move | 112 (24%) | 94 (89%) | 1.58 | 90 (88%) | 90 (88%) | 0 |
| Living alone | 56 (12%) | 9 (8%) | 16.5 | 6 (6%) | 6 (6%) | 0 |
| Absence of air conditioner | 54 (11%) | 18 (17%) | 10.7 | 16 (16%) | 15 (15%) | 2.7 |

We used a logistic regression model with six baseline independent variables (age, sex, underlying disease, inability to move, living alone, and absence of an air conditioner) to estimate propensity score. The receiver operating characteristic was 0.85 and the E-value was 1.4. For the calculation of E-values, see (1).

**Supplementary Table S3. Results from propensity score matching for absence of air-conditioning.**

|  | Unmatched groups | |  | Matched groups | |  |
| --- | --- | --- | --- | --- | --- | --- |
|  | have air-conditioners | have no air-conditioners |  | have air-conditioners | have no air-conditioners |  |
|  | n = 504 | n = 72 | SD (%) | n = 70 | n = 70 | SD (%) |
| Age (years) | 86.2 (7.2) | 84.5 (7.5) | 23.1 | 84.1 (6.6) | 84.7 (7.5) | 8.7 |
| Gender(male) | 228 (45%) | 38 (53%) | 15.1 | 38 (54%) | 38 (54%) | 0 |
| Underlying medical condition | 106 (21%) | 12 (17%) | 11.2 | 10 (14%) | 12(17%) | 7.9 |
| Inability to move | 176 (35%) | 30 (42%) | 78.6 | 24 (34%) | 28 (40%) | 11.8 |
| Living alone | 52 (10%) | 13 (18%) | 22.3 | 9 (13%) | 11 (16%) | 8.2 |
| Inability to drink water | 88 (17%) | 18 (25%) | 18.5 | 16 (23%) | 16 (23%) | 0 |

We used a logistic regression model with six baseline independent variables (age, sex, underlying disease, inability to move, living alone, and inability to drink water independently) to estimate propensity score. The receiver operating characteristic was 0.62 and the E-value was 1.8. For the calculation of E-values, see (1).

**Supplementary Table S4. List of variables, AICs, and MSEs for each prediction model using data from 2015 to 2019.**

| Factors of adaptation | Prediction models　no. |  | | Variables | | | | | | | |
| --- | --- | --- | --- | --- | --- | --- | --- | --- | --- | --- | --- |
|  |  | WBGT Threshold (℃) | The exponential rate of increase in risk | | the constant risk | Global solar radiation,  $s_{d}$ (kW/m2) | Extra warm days (definition) | Exceeding 31°C consecutive days, $u_{d}$ | Number of parameters | AIC | MSE |
| - | 1 | $T_{w}$ | $r$ | | $\beta$ |  |  |  | 3 | 7999.9 | 318.6 |
| Maximum daily average temperature | 2 | $T_{w.0}$ | $r_{0},r_{1}$ | | $\beta_{0}$^†^ | - | - | - | 4 | 7863.0 | 315.5 |
|  | 3 | $T_{w.0},T_{w.1}$ | $r_{0},r_{1}$ | | $\beta_{0}$^†^ | - | - | - | 5 | 7718.6 | 311.8 |
|  | 4 | $T_{w.0},T_{w.1}$ | $r_{0},r_{1}$ | | $\beta_{0},\beta_{1}$ | - | - | - | 6 | 7723.0 | 311.6 |
|  | 5 | $T_{w.0},T_{w.1}$ | $r_{0},r_{1}$ | | $\beta_{0}$^†^ | $\gamma_{1}$^†^ | - | - | 6 | 7127.9 | 277.8 |
|  | 6 | $T_{w.0},T_{w.1}$ | $r_{0},r_{1}$ | | $\beta_{0},\beta_{1}$ | $\gamma_{1}$^†^ | - | - | 7 | 7081.4 | 276.1 |
|  | 7 | $T_{w.0},T_{w.1}$ | $r_{0},r_{1}$ | | $\beta_{0}$^†^ | $\gamma_{1,0},\gamma_{1,1}$ | - | - | 7 | 7165.1 | 276.7 |
|  | 8 | $T_{w.0},T_{w.1}$ | $r_{0},r_{1}$ | | $\beta_{0},\beta_{1}$ | $\gamma_{1,0},\gamma_{1,1}$ | - | - | 8 | 7276.3 | 280.6 |
|  | 9 | $T_{w.0},T_{w.1}$ | $r_{0},r_{1}$ | | $\beta_{0}$^†^ | $\gamma_{1}$^†^ | 2 days | $\gamma_{2}$^†^ | *7* | 6781.3 | 243.2 |
|  | 10 | $T_{w.0},T_{w.1}$ | $r_{0},r_{1}$ | | $\beta_{0}$^†^ | $\gamma_{1}$^†^ | 2 days | $\gamma_{2,0},\gamma_{2,1}$ | *8* | 6666.1 | 241.8 |
|  | 11 | $T_{w.0},T_{w.1}$ | $r_{0},r_{1}$ | | $\beta_{0}$^†^ | $\gamma_{1}$^†^ | 3 days | $\gamma_{2}$^†^ | *7* | 6507.6 | 217.3 |
|  | 12 | $T_{w.0},T_{w.1}$ | $r_{0},r_{1}$ | | $\beta_{0}$^†^ | $\gamma_{1}$^†^ | 3 days | $\gamma_{2,0},\gamma_{2,1}$ | *8* | 6428.3 | 216.1 |
|  | 13 | $T_{w.0},T_{w.1}$ | $r_{0},r_{1}$ | | $\beta_{0},\beta_{1}$ | $\gamma_{1}$^†^ | 2 days | $\gamma_{2}$^†^ | *8* | 6693.6 | 246.0 |
|  | 14 | $T_{w.0},T_{w.1}$ | $r_{0},r_{1}$ | | $\beta_{0},\beta_{1}$ | $\gamma_{1}$^†^ | 2 days | $\gamma_{2,0},\gamma_{2,1}$ | *9* | 6652.7 | 241.1 |
|  | 15 | $T_{w.0},T_{w.1}$ | $r_{0},r_{1}$ | | $\beta_{0},\beta_{1}$ | $\gamma_{1}$^†^ | 3 days | $\gamma_{2}$^†^ | *8* | **6401.2** | 216.5 |
|  | 16 | $T_{w.0},T_{w.1}$ | $r_{0},r_{1}$ | | $\beta_{0},\beta_{1}$ | $\gamma_{1}$^†^ | 3 days | $\gamma_{2,0},\gamma_{2,1}$ | *9* | 6434.5 | 215.2 |
|  | 17 | $T_{w.0},T_{w.1}$ | $r_{0},r_{1}$ | | $\beta_{0}$^†^ | $\gamma_{1,0},\gamma_{1,1}$ | 2 days | $\gamma_{2}$^†^ | *8* | 6699.2 | 243.4 |
|  | 18 | $T_{w.0},T_{w.1}$ | $r_{0},r_{1}$ | | $\beta_{0}$^†^ | $\gamma_{1,0},\gamma_{1,1}$ | 2 days | $\gamma_{2,0},\gamma_{2,1}$ | *9* | 6720.6 | 242.0 |
|  | 19 | $T_{w.0},T_{w.1}$ | $r_{0},r_{1}$ | | $\beta_{0}$^†^ | $\gamma_{1,0},\gamma_{1,1}$ | 3 days | $\gamma_{2}$^†^ | *8* | 6432.9 | 216.9 |
|  | 20 | $T_{w.0},T_{w.1}$ | $r_{0},r_{1}$ | | $\beta_{0}$^†^ | $\gamma_{1,0},\gamma_{1,1}$ | 3 days | $\gamma_{2,0},\gamma_{2,1}$ | *9* | 6426.0 | 214.5 |
|  | 21 | $T_{w.0},T_{w.1}$ | $r_{0},r_{1}$ | | $\beta_{0},\beta_{1}$ | $\gamma_{1,0},\gamma_{1,1}$ | 2 days | $\gamma_{2}$^†^ | *9* | 6754.8 | 242.4 |
|  | 22 | $T_{w.0},T_{w.1}$ | $r_{0},r_{1}$ | | $\beta_{0},\beta_{1}$ | $\gamma_{1,0},\gamma_{1,1}$ | 2 days | $\gamma_{2,0},\gamma_{2,1}$ | *10* | 6754.2 | 241.8 |
|  | 23 | $T_{w.0},T_{w.1}$ | $r_{0},r_{1}$ | | $\beta_{0},\beta_{1}$ | $\gamma_{1,0},\gamma_{1,1}$ | 3 days | $\gamma_{2}$^†^ | *9* | 6460.5 | 216.6 |
|  | 24 | $T_{w.0},T_{w.1}$ | $r_{0},r_{1}$ | | $\beta_{0},\beta_{1}$ | $\gamma_{1,0},\gamma_{1,1}$ | 3 days | $\gamma_{2,0},\gamma_{2,1}$ | *10* | 6464.2 | 214.5 |

^1^AIC, Akaike information criterion; MSE, mean squared error. † The same parameter was used in the models after heat acclimation.

| Factors of adaptation | Prediction models　no. |  | | Variables | | | | | | | |
| --- | --- | --- | --- | --- | --- | --- | --- | --- | --- | --- | --- |
|  |  | WBGT Threshold (℃) | The exponential rate of increase in risk | | the constant risk | Global solar radiation,  $s_{d}$ (kW/m2) | Extra warm days (definition) | Exceeding 31°C consecutive days, $u_{d}$ | Number of parameters | AIC | MSE |
| - | 1 | $T_{w}$ | $r$ | | $\beta$ |  |  |  | 3 | - | - |
| Maximum daily average temperature | 2 | $T_{w.0}$ | $r_{0},r_{1}$ | | $\beta_{0}$^†^ | - | - | - | 4 | - | - |
|  | 3 | $T_{w.0},T_{w.1}$ | $r_{0},r_{1}$ | | $\beta_{0}$^†^ | - | - | - | 5 | - | - |
|  | 4 | $T_{w.0},T_{w.1}$ | $r_{0},r_{1}$ | | $\beta_{0},\beta_{1}$ | - | - | - | 6 | - | - |
|  | 5 | $T_{w.0},T_{w.1}$ | $r_{0},r_{1}$ | | $\beta_{0}$^†^ | $\gamma_{1}$^†^ | - | - | 6 | 8637.1 | 222.0 |
|  | 6 | $T_{w.0},T_{w.1}$ | $r_{0},r_{1}$ | | $\beta_{0},\beta_{1}$ | $\gamma_{1}$^†^ | - | - | 7 | 8598.0 | 222.7 |
|  | 7 | $T_{w.0},T_{w.1}$ | $r_{0},r_{1}$ | | $\beta_{0}$^†^ | $\gamma_{1,0},\gamma_{1,1}$ | - | - | 7 | 8701.3 | 223.3 |
|  | 8 | $T_{w.0},T_{w.1}$ | $r_{0},r_{1}$ | | $\beta_{0},\beta_{1}$ | $\gamma_{1,0},\gamma_{1,1}$ | - | - | 8 | 8811.6 | 225.1 |
|  | 9 | $T_{w.0},T_{w.1}$ | $r_{0},r_{1}$ | | $\beta_{0}$^†^ | $\gamma_{1}$^†^ | 2 days | $\gamma_{2}$^†^ | *7* | 8359.8 | 198.1 |
|  | 10 | $T_{w.0},T_{w.1}$ | $r_{0},r_{1}$ | | $\beta_{0}$^†^ | $\gamma_{1}$^†^ | 2 days | $\gamma_{2,0},\gamma_{2,1}$ | *8* | 8222.9 | 196.5 |
|  | 11 | $T_{w.0},T_{w.1}$ | $r_{0},r_{1}$ | | $\beta_{0}$^†^ | $\gamma_{1}$^†^ | 3 days | $\gamma_{2}$^†^ | *7* | **7944.2** | 178.8 |
|  | 12 | $T_{w.0},T_{w.1}$ | $r_{0},r_{1}$ | | $\beta_{0}$^†^ | $\gamma_{1}$^†^ | 3 days | $\gamma_{2,0},\gamma_{2,1}$ | *8* | 8035.9 | 175.9 |
|  | 13 | $T_{w.0},T_{w.1}$ | $r_{0},r_{1}$ | | $\beta_{0},\beta_{1}$ | $\gamma_{1}$^†^ | 2 days | $\gamma_{2}$^†^ | *8* | 8282.1 | 200.0 |
|  | 14 | $T_{w.0},T_{w.1}$ | $r_{0},r_{1}$ | | $\beta_{0},\beta_{1}$ | $\gamma_{1}$^†^ | 2 days | $\gamma_{2,0},\gamma_{2,1}$ | *9* | 8300.4 | 197.2 |
|  | 15 | $T_{w.0},T_{w.1}$ | $r_{0},r_{1}$ | | $\beta_{0},\beta_{1}$ | $\gamma_{1}$^†^ | 3 days | $\gamma_{2}$^†^ | *8* | 7962.7 | 178.7 |
|  | 16 | $T_{w.0},T_{w.1}$ | $r_{0},r_{1}$ | | $\beta_{0},\beta_{1}$ | $\gamma_{1}$^†^ | 3 days | $\gamma_{2,0},\gamma_{2,1}$ | *9* | 7991.1 | 176.2 |
|  | 17 | $T_{w.0},T_{w.1}$ | $r_{0},r_{1}$ | | $\beta_{0}$^†^ | $\gamma_{1,0},\gamma_{1,1}$ | 2 days | $\gamma_{2}$^†^ | *8* | 8252.4 | 197.8 |
|  | 18 | $T_{w.0},T_{w.1}$ | $r_{0},r_{1}$ | | $\beta_{0}$^†^ | $\gamma_{1,0},\gamma_{1,1}$ | 2 days | $\gamma_{2,0},\gamma_{2,1}$ | *9* | 8274.8 | 196.1 |
|  | 19 | $T_{w.0},T_{w.1}$ | $r_{0},r_{1}$ | | $\beta_{0}$^†^ | $\gamma_{1,0},\gamma_{1,1}$ | 3 days | $\gamma_{2}$^†^ | *8* | 7991.8 | 177.8 |
|  | 20 | $T_{w.0},T_{w.1}$ | $r_{0},r_{1}$ | | $\beta_{0}$^†^ | $\gamma_{1,0},\gamma_{1,1}$ | 3 days | $\gamma_{2,0},\gamma_{2,1}$ | *9* | 7982.2 | 175.7 |
|  | 21 | $T_{w.0},T_{w.1}$ | $r_{0},r_{1}$ | | $\beta_{0},\beta_{1}$ | $\gamma_{1,0},\gamma_{1,1}$ | 2 days | $\gamma_{2}$^†^ | *9* | 8317.0 | 197.8 |
|  | 22 | $T_{w.0},T_{w.1}$ | $r_{0},r_{1}$ | | $\beta_{0},\beta_{1}$ | $\gamma_{1,0},\gamma_{1,1}$ | 2 days | $\gamma_{2,0},\gamma_{2,1}$ | *10* | 8324.0 | 196.8 |
|  | 23 | $T_{w.0},T_{w.1}$ | $r_{0},r_{1}$ | | $\beta_{0},\beta_{1}$ | $\gamma_{1,0},\gamma_{1,1}$ | 3 days | $\gamma_{2}$^†^ | *9* | 8045.9 | 178.5 |
|  | 24 | $T_{w.0},T_{w.1}$ | $r_{0},r_{1}$ | | $\beta_{0},\beta_{1}$ | $\gamma_{1,0},\gamma_{1,1}$ | 3 days | $\gamma_{2,0},\gamma_{2,1}$ | *10* | 8023.7 | 176.5 |

**Supplementary Table S5. List of variables, AICs, and MSEs for each prediction model using data from 2013 to 2019.**

^1^AIC, Akaike information criterion; MSE, mean squared error. † The same parameter was used in the models after heat acclimation.

We used data from 2013 to 2019, with data collected between May and September for 2015 to 2019, and between June and September for 2013 and 2014. The reason for the variation in the months of data collection by year is due to changes in the method of data collection for official records.

In Model 15, maximum likelihood estimates of the parameter were estimated at *T_w,0_* = 21.8, *T_w,1_* = 19.8, *r_0_* = 0.31, *r_1_* = 0.34, *β_0_* = 0.75, *β_1_* = 0.26, *γ_1_* = 0.05, and *γ_2_* = 0.52, respectively.

**Supplementary Table S6. Relative risk of the increase in heatstroke-related ambulance transports relative to 5-year median from 2015-19 and carbon-neutral scenario** **: 2013-2019 Parameter Estimation.**

| scenario | baseline |  | 2030s | 2040s | 2050s | 2060s | 2070s | 2080s | 2090s | 2100 |
| --- | --- | --- | --- | --- | --- | --- | --- | --- | --- | --- |
| MIROC6 | 2015-19 | RCP2.6 | 38.0 (27, 44.9) | 26.9 (8.4, 34.9) | 26.9 (4.7, 29.5) | 9.2 (0, 34.2) | 14.9 (0.6, 29.1) | 22.6 (9.7, 36.7) | 38.1 (0, 44.9) | 7.5 |
|  |  | RCP4.5 | 32.1 (22.7, 44) | 13.6 (0, 33.3) | 31 (13.8, 43.4) | 27.8 (12.6, 38.1) | 38.2 (27.6, 42.3) | 37.2 (33.6, 53.2) | 50.2 (41.8, 59.6) | 53.1 |
|  |  | RCP8.5 | 31.1 (13.8, 40.5) | 13.8 (0, 26.3) | 41.8 (20.9, 46) | 52.8 (34.3, 58.3) | 60.6 (52.6, 63.8) | 63.9 (54.7, 66.4) | 66.1 (63.5, 73.9) | 74.5 |
|  | RCP1.9 | RCP2.6 | 6.4 (0, 27.5) | 4.6 (0, 18.5) | 0.1 (0, 22.4) | 7.1 (0, 42) | 0 (0, 35.1) | 10.6 (0, 19.8) | 3.1 (0, 15.1) | 0 |
|  |  | RCP4.5 | 1.4 (0, 24.2) | 0 (0, 10.5) | 4.6 (0, 30.6) | 26.4 (0, 45.3) | 24.2 (0.6, 47.9) | 24.9 (8.3, 39.3) | 15 (0, 43.5) | 13 |
|  |  | RCP8.5 | 0 (0, 18.4) | 0 (0, 4.5) | 13.1 (1.8, 39.6) | 52.7 (21.2, 62.2) | 53.9 (42.8, 61.1) | 53.3 (46.3, 63.1) | 47.7 (38.4, 52.8) | 52.7 |
| MRI-ESM-2.0 | 2015-19 | RCP2.6 | 34.7 (10.3, 44.1) | 12.3 (0, 35.4) | 30.7 (24.5, 34.8) | 32.6 (21.2, 42.1) | 30.5 (17.6, 47.4) | 39.8 (30.4, 46.8) | 21.7 (14, 45) | 10.6 |
|  |  | RCP4.5 | 23.1 (17.2, 33.3) | 22.4 (11, 33.9) | 28.6 (18.3, 35.8) | 41.6 (32.7, 47) | 43.3 (28.4, 55.3) | 46.2 (34, 55.1) | 52.8 (43.3, 61.1) | 47 |
|  |  | RCP8.5 | 40.7 (29.1, 48.4) | 28 (7.9, 42.8) | 32.6 (25.7, 36.3) | 52.5 (40.8, 60.5) | 46.3 (42.3, 50.9) | 56.5 (48.3, 64.2) | 65.4 (55.5, 69) | 65.5 |
|  | RCP1.9 | RCP2.6 | 6.2 (0, 14.3) | 0 (0, 23.5) | 7.7 (4.2, 17.4) | 13.8 (0, 27) | 4.3 (0, 35.4) | 19.2 (10.7, 34.3) | 0.2 (0, 16.8) | 0 |
|  |  | RCP4.5 | 0 (0, 3.9) | 0 (0, 12.6) | 7.1 (0, 20.6) | 24.8 (8.3, 36.5) | 13.2 (0, 43.6) | 30.5 (22.7, 41.7) | 37.6 (17.2, 52.9) | 33.8 |
|  |  | RCP8.5 | 11.2 (0, 24.7) | 0 (0, 15.5) | 10.4 (2, 19.1) | 38.7 (21, 52.7) | 22.4 (14.5, 39.6) | 43.3 (37.4, 52.6) | 53.4 (35.6, 63.8) | 56.8 |
| IPSL | 2015-19 | RCP2.6 | 15.2 (0, 28.9) | 7.3 (0, 26.1) | 0 (0, 29.9) | 11.3 (0, 27.3) | 28.5 (19.5, 37.8) | 20.9 (15.1, 27.6) | 30.2 (9.9, 39) | 27.2 |
|  |  | RCP4.5 | 22.3 (8, 34.2) | 12.8 (0, 21.4) | 11.8 (6.3, 20.5) | 29.5 (24.5, 34.8) | 34.1 (26.1, 50.2) | 53.8 (46.1, 61.1) | 49.9 (42.4, 53.5) | 48.7 |
|  |  | RCP8.5 | 7.6 (0, 32.4) | 29.3 (18.3, 40.2) | 28.2 (7.9, 55) | 57.4 (46.9, 62.2) | 57 (54.6, 63.9) | 69.6 (59.4, 74.1) | 76.6 (75.4, 77.8) | 71.9 |
|  | RCP1.9 | RCP2.6 | 3.9 (0, 20.8) | 9.7 (0, 21.2) | 0 (0, 31.9) | 24 (0, 40.1) | 8.5 (0, 22.6) | 28.7 (18.8, 40.7) | 21.9 (0, 40.6) | 16.6 |
|  |  | RCP4.5 | 8 (0, 28.6) | 3.9 (0, 31.1) | 11.5 (0, 27.1) | 37.6 (1, 47.5) | 17.4 (5.9, 37.6) | 57.5 (39.6, 70.6) | 44.1 (39, 55.3) | 41.3 |
|  |  | RCP8.5 | 0 (0, 8.4) | 24.7 (10.7, 41.1) | 34.1 (20.3, 43.6) | 60.9 (30, 70.8) | 45.1 (42.8, 54.4) | 73.5 (54.5, 80.5) | 74.4 (71.4, 77.4) | 67.9 |

**Supplementary Table S7. The incidence rate of transported heatstroke among 65 years old and older in Japan.**

|  | 2015 | 2016 | 2017 | 2018 | 2019 |
| --- | --- | --- | --- | --- | --- |
| The population of all ages  (Thousand persons) | 127095 | 125020 | 124648 | 124218 | 126167 |
| The population of 65+  (Thousand persons) | 33869 | 34451 | 35007 | 35427 | 35885 |
| Transported heatstroke cases  (cases) | 55852 | 50412 | 52984 | 95137 | 71317 |
| Transported heatstroke cases of 65+  (cases) | 28016 | 25228 | 25930 | 45781 | 37091 |
| The incidence rate of heatstroke in those 65+  (%) | 0.08 | 0.07 | 0.07 | 0.13 | 0.10 |
| The proportion of heatstroke in those 65+  (%) | 50.2 | 50.0 | 48.9 | 48.1 | 52.0 |

We collected population data from the national census records. “Transported heatstroke cases” refers to the number of heatstroke patients transported between May and September, based on publicly available data.

# Reference

1. Ioannidis JPA, Tan YJ, Blum MR. Limitations and misinterpretations of E-values for sensitivity analyses of observational studies. Ann Intern Med (2019) 170:108–11. doi: 10.7326/M18-2159
